# Clarin‐1 expression in adult mouse and human retina highlights a role of Müller glia in Usher syndrome

**DOI:** 10.1002/path.5360

**Published:** 2019-12-04

**Authors:** Lei Xu, Susan N Bolch, Clayton P Santiago, Frank M Dyka, Omar Akil, Ekaterina S Lobanova, Yuchen Wang, Kirill A Martemyanov, William W Hauswirth, W Clay Smith, James T Handa, Seth Blackshaw, John D Ash, Astra Dinculescu

**Affiliations:** ^1^ Department of Ophthalmology University of Florida Gainesville FL USA; ^2^ Solomon H Snyder Department of Neuroscience Johns Hopkins University, School of Medicine Baltimore MD USA; ^3^ Department of Otolaryngology–HNS University of California San Francisco CA USA; ^4^ Department of Neuroscience The Scripps Research Institute Jupiter FL USA; ^5^ Department of Ophthalmology Johns Hopkins University School of Medicine Baltimore MD USA; ^6^ Department of Neurology Johns Hopkins University School of Medicine Baltimore MD USA; ^7^ Center for Human Systems Biology Johns Hopkins University School of Medicine Baltimore MD USA; ^8^ Institute for Cell Engineering, Johns Hopkins University School of Medicine Baltimore MD USA; ^9^ Kavli Neuroscience Discovery Institute Johns Hopkins University School of Medicine Baltimore MD USA

**Keywords:** Usher syndrome pathology, Müller glia, human retina, clarin‐1 expression, retinal degeneration, transcriptomic analysis

## Abstract

Usher syndrome type 3 (USH3) is an autosomal recessively inherited disorder caused by mutations in the gene clarin‐1 (*CLRN1*), leading to combined progressive hearing loss and retinal degeneration. The cellular distribution of CLRN1 in the retina remains uncertain, either because its expression levels are low or because its epitopes are masked. Indeed, in the adult mouse retina, *Clrn1* mRNA is developmentally downregulated, detectable only by RT‐PCR. In this study we used the highly sensitive RNAscope *in situ* hybridization assay and single‐cell RNA‐sequencing techniques to investigate the distribution of *Clrn1* and *CLRN1* in mouse and human retina, respectively. We found that *Clrn1* transcripts in mouse tissue are localized to the inner retina during postnatal development and in adult stages. The pattern of *Clrn1* mRNA cellular expression is similar in both mouse and human adult retina, with *CLRN1* transcripts being localized in Müller glia, and not photoreceptors. We generated a novel knock‐in mouse with a hemagglutinin (HA) epitope‐tagged CLRN1 and showed that CLRN1 is expressed continuously at the protein level in the retina. Following enzymatic deglycosylation and immunoblotting analysis, we detected a single CLRN1‐specific protein band in homogenates of mouse and human retina, consistent in size with the main CLRN1 isoform. Taken together, our results implicate Müller glia in USH3 pathology, placing this cell type to the center of future mechanistic and therapeutic studies to prevent vision loss in this disease. © 2019 The Authors. *The Journal of Pathology* published by John Wiley & Sons Ltd on behalf of Pathological Society of Great Britain and Ireland.

## Introduction

Usher syndrome (USH) represents a group of autosomal recessive disorders, characterized by combined vision and hearing loss, and in some cases vestibular dysfunction 1. The disease primarily affects the light‐absorbing photoreceptor neurons in the retina and the auditory hair cells in the inner ear, ultimately causing their progressive death. It is grouped into three subtypes (USH1, USH2, and USH3) based on its diverse clinical symptoms, particularly the severity of hearing impairment. USH3 is characterized by a progressive hearing loss and variable onset and severity of retinal degeneration 2. It is caused by mutations in the gene clarin‐1 (*CLRN1*), whose main isoform encodes a 232‐amino acid protein predicted to have four transmembrane domains, and a single glycosylation site in the first extracellular loop 3. In the cochlea, CLRN1 is involved in the morphogenesis and maintenance of hair bundle stereocilia and it may also have synaptic roles 4, 5, 6, 7, 8. *In vitro* biochemical assays suggest that CLRN1 functions as a molecular scaffold, recruiting proteins involved in cell adhesion at distinct plasma membrane regions and playing a role in organizing the actin cytoskeleton 9. Consistent with this function, *Clrn1* knockout (KO) and N48K knock‐in mice display poorly developed and disorganized F‐actin‐rich stereocilia at a young age, and are profoundly deaf by postnatal day 21 (P21) 5, 9, 10, 11. However, similar to other mouse models of USH disease, these mice do not mimic the ocular phenotype found in USH3 patients, and display no retinal degeneration 11, 12, 13.

The function of CLRN1 in the retina is currently unknown, primarily due to the lack of appropriate USH3 animal models and a major gap in our knowledge regarding its cellular localization. Three previous studies focusing on localizing CLRN1 in the retina have yielded conflicting results 11, 14, 15. In one study, *Clrn1* mRNA in the mouse retina was shown to have the highest expression in the early postnatal retina, and was detected exclusively in the inner nuclear layer (INL) by *in situ* hybridization 11. In adult stages, *Clrn1* mRNA was detectable only by RT‐PCR and remained confined to the inner retina. During postnatal development, *Clrn1* transcripts in the INL were found to co‐localize with Müller cell‐specific markers, suggesting that in the retina, *Clrn1* was expressed primarily in Müller glia cells. Another group reported that CLRN1 protein was expressed in mouse photoreceptors, in synaptic and connecting cilium regions 14. In zebrafish, CLRN1 protein was detected both in photoreceptors and in the inner retina 15. CLRN1 protein detection by western blotting was also reported, with bands ranging in size from 25 to 50 kDa, but the interpretation of these results was hampered by the lack of negative controls 11, 15. The cellular localization of CLRN1 remains uncertain because numerous studies were unable to detect this protein *in situ*, leading some investigators to suggest that its levels are very low, or its epitopes are masked 5, 6, 11, 16.

The phenotypic discrepancy between the mouse and human USH3 retinal phenotype, combined with the knowledge that *Clrn1* mRNA is developmentally downregulated in mouse retina and the documented repeated failed attempts to reliably detect retinal CLRN1 protein, raises a number of fundamental questions. Is the cellular pattern of *Clrn1* mRNA expression in the mouse retina different from human? Is CLRN1 protein in mouse retina present only transiently during development, or does it exhibit continuous expression into adulthood? Do human and mouse retinas express distinct CLRN1 isoforms at the protein level? The answers to these questions are critical for designing adeno‐associated virus (AAV)‐based treatment strategies for therapeutic interventions to prevent vision loss in USH3 patients and may also provide clues for understanding the differences in the ocular phenotype between mouse models and human USH3. Therefore, in the current study we examined the pattern of CLRN1 expression in mouse and human retina by using a combination of novel tools, including the highly sensitive RNAscope ISH technique for visualizing *Clrn1* transcripts in tissue sections and single‐cell RNA sequencing (scRNA‐seq) analysis to identify the specific cell types in which *Clrn1* mRNA is expressed. Interestingly, our findings reveal that *Clrn1* transcripts in both mouse and human adult retinas are concentrated in the INL, being specifically expressed in Müller glia, and not in photoreceptor cells. In addition, to overcome the existing challenges in reliably detecting CLRN1 protein expression, we generated and characterized a novel knock‐in mouse with an N‐terminal hemagglutinin (HA) epitope‐tagged endogenous CLRN1. We found that although *Clrn1* mRNA is developmentally downregulated, CLRN1 protein is expressed continuously during adulthood in the mouse. Following enzymatic deglycosylation, a single CLRN1 protein product was detected by immunoblotting in both mouse and human retinal extracts, consistent in size with the main CLRN1 isoform. This study shifts our understanding of the pathological mechanisms in USH3 in a novel direction, one that requires the study of glia–neuron interactions in the human retina, instead of focusing exclusively on photoreceptor neurons. Our results provide important fundamental knowledge for developing therapeutic approaches to prevent vision loss in USH3, and for future studies aimed at uncovering the mechanisms by which *CLRN1* mutations lead to photoreceptor degeneration.

## Materials and methods

### Animal care and use

All animal procedures, maintenance, and handling were approved by the University of Florida Institutional Animal Care and Use Committee (IACUC) and conducted in accordance with the Association for Research in Vision and Ophthalmology (ARVO) Statement for the Use of Animals in Ophthalmic and Vision Research (http://www.arvo.org/About/policies/statement-for-the-use-of-animals-in-ophthalmic-and-vision-research/). Wild‐type C57BL/6J mice served as controls and were purchased from the Jackson Laboratory (Bar Harbor, ME, USA). The N‐HA‐tagged *Clrn1* knock‐in mouse contains a 30‐nucleotide sequence (TAC CCA TAC GAT GTT CCA GAT TAC GCT GAG) at the 5′‐end of *Clrn1* (NM_153385.3), immediately after the ATG start codon. This mouse was generated at the Jackson Laboratory by using the CRISPR/Cas9 technology in the C57BL/6J strain as described in supplementary material, Supplementary materials and methods.

### RNAscope *in situ* hybridization

*In situ* hybridization (ISH) was performed on formalin‐fixed, paraffin embedded sections as directed by the manufacturer (Advanced Cell Diagnostics, Hayward, CA, USA). We used RNAscope LS 2.5 Probe – Mm‐Clrn1 (catalog # 400178) to detect mouse *Clrn1* and RNAscope® 2.5 LS Probe – Hs‐CLRN1 (catalog # 562958) to detect human *CLRN1*. The *Clrn1* mouse probe was designed to span exons 1, 3, and 4 of the main *Clrn1* isoform, and included the region comprising nucleotides 106–1146 of mouse *Clrn1* (NM_153385.2). The human probe included the region 325–2352 of human *CLRN1* (NM_174878.2). Both probes were designed to detect all validated mRNA isoforms. Positive control sections detected reference transcripts encoding RNA polymerase II subunit A (RNAscope 2.5 LS Probe – Mm‐Polr2a, catalog # 312478 for mouse, and RNAscope® 2.5 LS Probe Hs‐POLR2A, catalog # 310458 for human, respectively). Negative control sections were probed for bacterial dihydrodipicolinate reductase mRNA (dapB). Detailed information on the ISH procedure can be found in supplementary material, Supplementary materials and methods.

### Single‐cell RNA sequencing analysis

*Clrn1/CLRN1* transcripts were analyzed in mouse, non‐human primate (NHP), and human retinas. The data for NHP were a reanalysis from a previously reported database 17. The human scRNA‐seq data were generated at John Hopkins University. Single‐cell libraries were prepared as described previously 18. Cell Ranger matrix files were analyzed using Seurat to re‐cluster and identify cell types using known cell‐specific markers, and to generate violin/scatter plots of *CLRN1* gene expression data in known cell types 19. Detailed information can be found in supplementary material, Supplementary materials and methods.

### Immunoblotting analyses

Details on processing human and mouse retina samples are described in supplementary material, Supplementary materials and methods. A rabbit monoclonal anti‐HA antibody (catalog # 3724S, clone C29F4, 1:3000; Cell Signaling Technology, Danvers, MA, USA) was used to detect the N‐HA‐tagged CLRN1 protein; an anti‐CLRN1 rabbit polyclonal antibody (catalog # 26630‐1‐AP, 1:2000; Proteintech, Rosemont, IL, USA) was used to detect human CLRN1; and mouse monoclonal anti‐alpha tubulin (catalog # T5168, clone B‐5‐1‐2, 1:5000, Millipore Sigma, Burlington, MA, USA) served as a loading control antibody.

### Immunohistochemistry

Different tissue preparation methods and antibodies were used for CLRN1‐HA detection, as described in supplementary material, Supplementary materials and methods.

### Statistical analysis

GraphPad Prism version 8 (GraphPad Software, San Diego, CA, USA) was used for statistical analysis. Differences between groups were analyzed either by one‐way analysis of variance (ANOVA) with Tukey's *post hoc* comparisons or by a two‐sided Student's *t*‐test (where only two groups were analyzed). Graphs show mean ± SEM; *P* values less than 0.05 were considered statistically significant.

## Results

### mRNA expression in mouse and human retina

**Figure 1:**
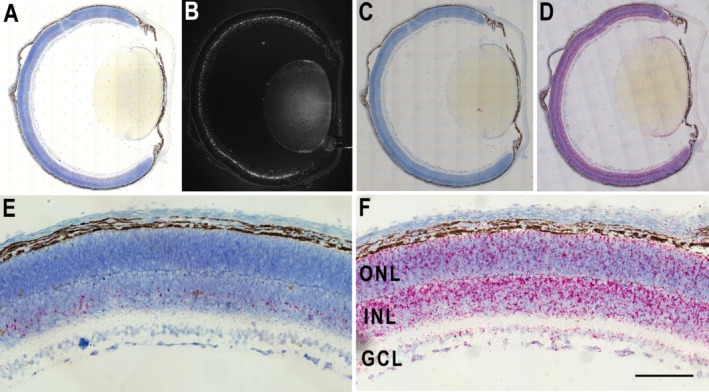
*Clrn1* mRNA expression pattern in the mouse eye. (A) *In situ* hybridization using sections of mouse eye from postnatal day 8 (P8) probed with a *Clrn1*‐specific probe. The signal appears as red punctate staining throughout the inner nuclear layer (INL). (B) The same section as in panel A, imaged by fluorescence microscopy (greyscale image). (C) P8 mouse eye section probed for dapB as a negative control. (D) P8 eye section showing the positive control polymerase II subunit mRNA expression (*Polr2A* probe). (E) Representative image (enlarged view) showing *Clrn1* mRNA expression in the INL. (F) *Polr2a* positive control mRNA expression (enlarged view). Cell nuclei are stained with hematoxylin. ONL, outer nuclear layer; INL, inner nuclear layer; GCL, ganglion cell layer. Scale bar = 50 μm.

In order to visualize the expression pattern of *Clrn1* transcripts in the intact retinal tissue, we used RNAscope chromogenic ISH on sections of mouse and human retinas 20. We first examined the pattern of *Clrn1* mRNA expression in the mouse retina during postnatal development and adult stages, either under standard bright‐field or by fluorescence microscopy (Figure 1 and supplementary material, Figure S1). *Clrn1* transcripts were detected in the inner retina and were absent from photoreceptors, in agreement with previous findings 11. The signal was weakly present at P4 and increased in intensity up until P14, after which it slowly declined to lower levels found in adult retinas. By P90, the punctate ISH staining in the inner retina became more restricted and sparse, indicating that the levels of *Clrn1* mRNA transcripts are much lower in adulthood. Interestingly, we found that *Clrn1* was also expressed in the lens (Figure 1A,B). This may correlate with clinical findings in USH patients, noting a higher risk of developing cataracts 21, 22. No labeling of RPE cells was detected, indicating that *Clrn1* mRNA is not expressed in these cells. The bacterial dihydrodipicolinate reductase probe (dapB) used as a negative control produced no signal (Figure 1C). The murine RNA polymerase II subunit A probe (*Polr2A*) used as a positive control generated an abundant signal in all the layers of the retina, as expected (Figure 1D). No signal was detected when the same *Clrn1* probe was used in *Clrn1* KO sections (supplementary material, Figure S2). We also stained the same retinal sections from ISH experiments with an antibody to glutamine synthetase (GS), which specifically labels Müller glial cells. The co‐localization of *Clrn1* transcripts to the same cell bodies expressing GS in the INL suggests that Müller cells in mouse retina express *Clrn1* (supplementary material, Figure S3).

**Figure 2:**
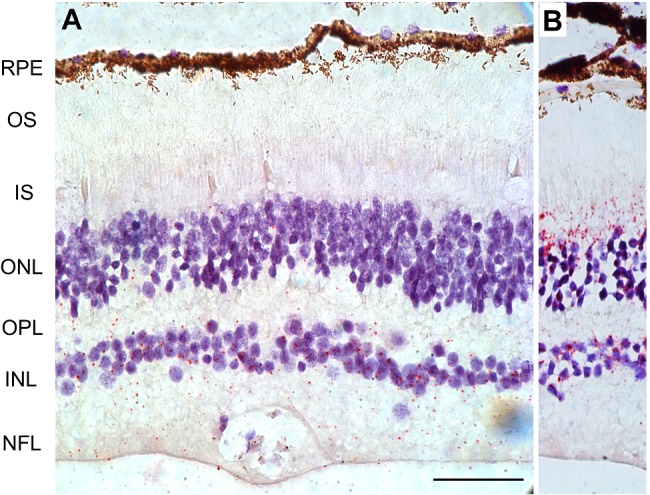
Localization of *CLRN1* transcripts in adult human retina by ISH. (A) Detection of *CLRN1* mRNA with a human *CLRN1*‐specific probe on a section of paraformaldehyde‐fixed, paraffin‐embedded human retina. Note the red punctate ISH signals within the INL and extending towards the NFL. (B) Expression of the human RNA polymerase II subunit A (*POLR2A*) as a positive control. RPE, retinal pigmented epithelium; OS, outer segment; IS, inner segment; ONL, outer nuclear layer; OPL, outer plexiform layer; INL, inner nuclear layer; NFL, nerve fiber layer. Scale bar = 50 μm.

We examined *CLRN1* mRNA expression in the adult human retina (Figure 2 and supplementary material, Figure S4). Transcripts were primarily concentrated in the INL, with occasional punctate signals extending towards the nerve fiber layer (NFL; Figure 2). In addition, we noticed the presence of sparse, punctate ISH chromogenic labeling of *CLRN1* mRNA in the outer nuclear layer (ONL) and outer limiting membrane (OLM) that may result from Müller cell processes extending into those regions 23. Previous studies have indeed shown that the mRNA in Müller glia can be spatially distributed within radially oriented processes over the entire thickness of the retina 24.

**Figure 3:**
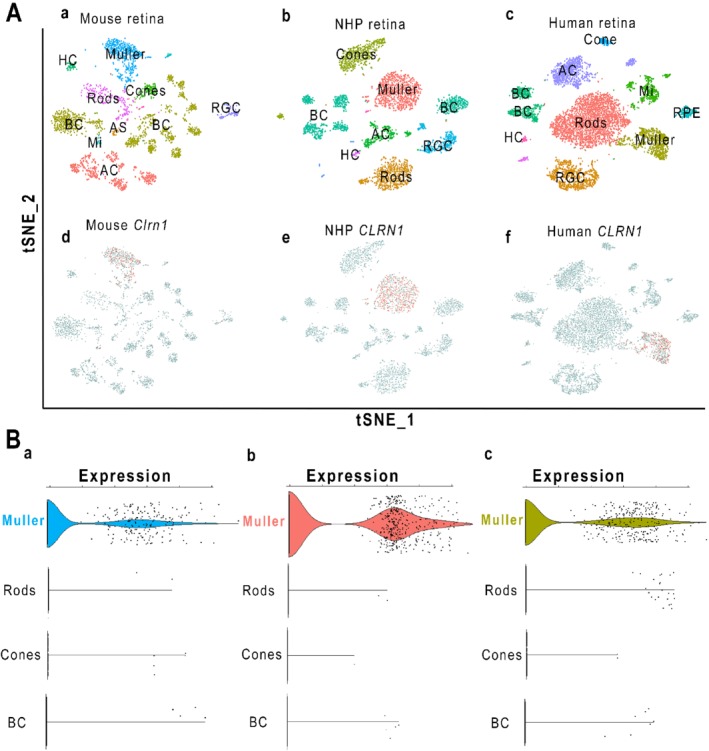
Single‐cell RNAseq analysis of *Clrn1* expression in mouse, NHP, and human retina. (A) T‐distributed stochastic neighbor embedding (t‐SNE) was used to cluster cells. Each dot represents a single cell. The t‐SNE plots place cells on the graph based on the expression of highly variable genes. This is a method that places similar cells in distinct clusters. Cells in each cluster were identified based on the expression of known cell markers listed in the Materials and methods section (a–c). Cells expressing *Clrn1* are shown in red and non‐expressing cells are grey (d–f). *Clrn1*‐positive cells were found overlapping with Müller glia clusters in all three species. Cell populations are Müller glia (Muller), rods, cones, retinal ganglion cells (RGC), horizontal cells (HC), bipolar cells (BC), amacrine cells (AC), microglia (Mi), vascular endothelial (VE), retinal pigment epithelium (RPE), and astrocytes (AS). (B) Using Seurat, we extracted the expression of *Clrn1* in all cell clusters. The scatter plots in B show cells in each cluster that are positive for *Clrn1* in mouse (a), NHP (b), and human retinas (c). The violin plots show cell types with *Clrn1‐*enriched expression. Shown here are cell populations including Müller glia, rods, cones, and bipolar cells (BC).

To reliably identify retinal cell types that express *Clrn1* mRNA, we used single‐cell RNA‐seq (scRNA‐seq) transcriptomic analysis on retinas from mice, macaque NHP, and humans. In order to identify and cluster retinal cell types, we used the t‐distributed stochastic neighbor embedding (t‐SNE) principal component method in Seurat to visualize cells that are plotted in clusters based on highly variant gene expression 19. Cells with similar gene expression patterns are clustered in distinct groups. Known cell‐specific markers are then used to identify major retinal cell types (Figure 3A). We then identified which cell clusters expressed *Clrn1*. In all three species, *Clrn1*‐expressing cells co‐localized with Müller cells (Figure 3A). Expression levels of *Clrn1* were extracted from the data of all cells, and as shown in Figure 3B and supplementary material, Figure S5, Müller cells in all three species have statistically enriched expression of *Clrn1*, as reflected by the violin plots. The enriched expression in Müller cells is consistent with the ISH localization of *Clrn1* mRNA described above. No other cell type demonstrated this enrichment.

### Generation and characterization of a novel epitope‐tagged *Clrn1* knock‐in mouse

To overcome challenges in detecting CLRN1 protein in the retina, we generated and characterized an epitope‐tagged *Clrn1* knock‐in mouse by inserting a small HA epitope at the N‐terminus of CLRN1 (supplementary material, Figure S6). By using this knock‐in approach, the HA‐tagged CLRN1 remains under the control of its natural promoter and other regulatory elements, and therefore the expression of different splice variants is preserved at physiologically relevant levels. Moreover, this knock‐in mouse enables us to bypass the use of anti‐CLRN1 antibodies, and to differentiate specific HA‐CLRN1 immunoreactive bands from background or cross‐reacting signals, even if CLRN1 was expressed at low levels, by using a monoclonal, high‐affinity anti‐HA antibody and C57BL/6J retinal extracts as negative controls. RT‐PCR was first used to evaluate the expression of different *Clrn1* isoforms (supplementary material, Figure S6C). The *CLRN1* gene contains four exons, and multiple mRNA splice variants are thought to exist 25. All known USH3‐associated mutations are found in isoform 2 (main *CLRN1* isoform, containing exons 1, 3, and 4), encoding a 232‐amino acid protein. By using the F1 primer against exon 1, we amplified two bands, which sequencing identified as the main isoform 2 and the shorter isoform 3, containing exons 1 and 4, and encoding a 172‐amino acid CLRN1 protein. This result is consistent with previous studies, showing that these two transcripts are primarily amplified from retinal cDNA using primers that span all four *Clrn1* exons 11.

**Figure 4:**
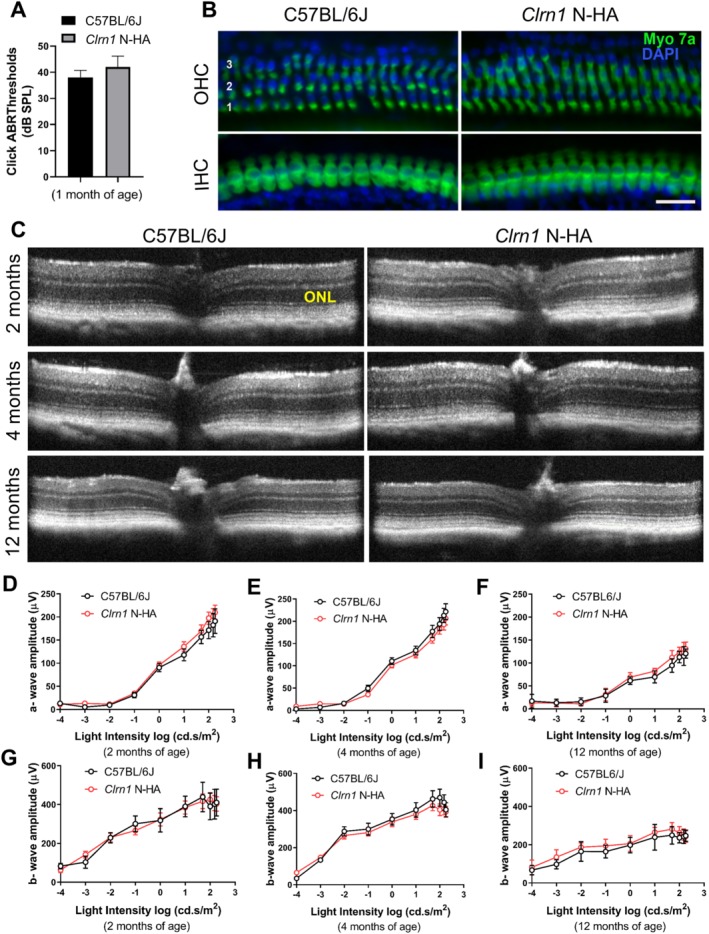
Characterization of the *Clrn1* N‐HA knock‐in mouse model compared with C57BL/6J. (A) Auditory function in WT and *Clrn1* N‐HA mice. Acoustic brainstem response (ABR) testing of broadband clicks stimuli at P30 demonstrates no significant differences in hearing between the WT (*n* = 6) and N‐HA‐*Clrn1* knock‐in mice (*n* = 6). (B) Cochlear whole‐mount immunofluorescence staining for myosin 7a (a marker of cochlear hair cells). Outer hair cells (OHC) rows 1–3 are indicated by 1, 2, and 3, respectively. IHC: inner hair cells. Scale bar = 30 μm. (C) SD‐OCT imaging showing normal retinal structure in the N‐HA‐*Clrn1* knock‐in mice (*n* = 5). (D–I) Retinal function measured by scotopic ERG at 2, 4, and 12 months of age. There were no significant differences in the N‐HA‐*Clrn1* knock‐in mice when compared with C57BL/6J mice up to 12 months of age (*n* = 5–8 mice).

In contrast to an early‐onset hearing loss, genetic mouse models of USH3 do not mimic the retinal degeneration found in USH3 patients 11, 12. Both *Clrn1* KO and N48K knock‐in mice are deaf by P21 but display normal retinal function and morphology throughout their lifetime when raised on a C57BL/6J background. Therefore, any potential detrimental effects of the HA tag on CLRN1 protein function will most likely impact the hearing and hair cell morphology at early stages during development. Consequently, we first tested if the presence of the HA tag results in the loss of hearing function in homozygous HA‐tagged *Clrn1* knock‐in mice. We performed acoustic brainstem response (ABR) testing of broadband clicks stimuli on N‐HA *Clrn1* knock‐in mice at P30 compared with age‐matched C57BL/6J controls. We did not observe any significant differences in hearing between the two groups (Figure 4A). We also performed immunofluorescence staining using an antibody against myosin 7a (Myo7a) to examine whether the overall morphology of cochlear hair cells in the N‐HA *Clrn1* knock‐in mice was normal compared with wild‐type controls. Hair cell counts using representative samples of surface preparations showed a normal complement of inner (IHCs) and outer hair cells (OHCs), with no IHC or OHC loss at any locations along the cochlear turns (Figure 4B). In order to examine if retina thickness was affected in the N‐HA‐tagged *Clrn1* knock‐in mice, we employed a non‐invasive technique, spectral‐domain optical coherence tomography (SD‐OCT). We did not observe any significant changes in structure of the retina at 2, 4 or 12 months of age (Figure 4C). To determine if the presence of the HA tag could potentially affect retinal function, we next performed periodic electroretinography (ERG) evaluations on a group of homozygous N‐HA‐tagged *Clrn1* knock‐in mice up to 12 months of age, and did not detect any significant differences compared with C57BL/6J controls (Figure 4D–I). These experiments demonstrated that retinal function and hearing are normal in the N‐HA‐tagged *Clrn1* knock‐in mice, suggesting that placement of the HA tag at the N‐terminal end of the CLRN1 protein did not interfere with its biological roles.

### Detection of glycosylated CLRN1 protein in mouse and human retina

**Figure 5:**
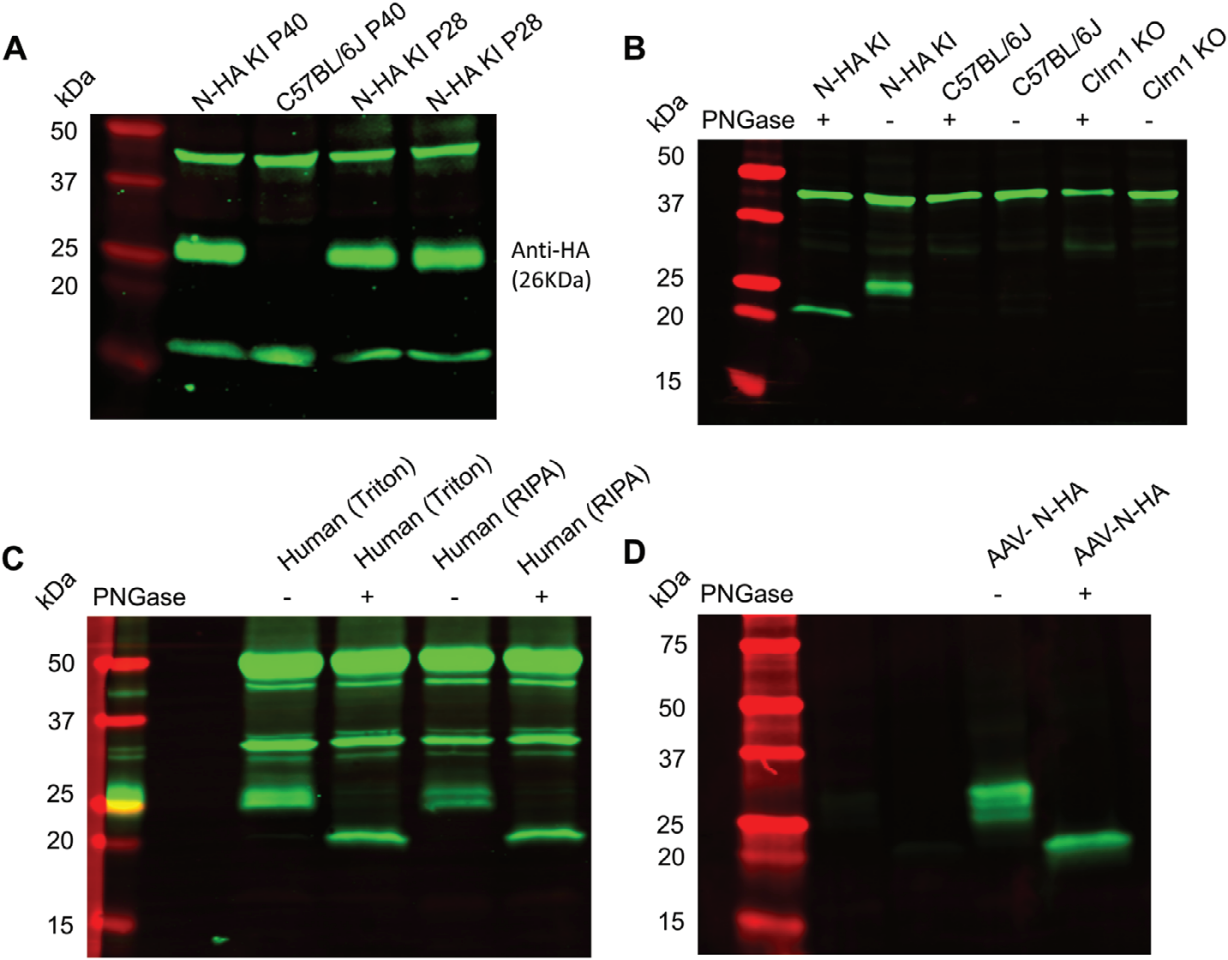
Detection of the HA‐tagged CLRN1 protein by immunoblotting. (A) Representative immunoblots showing the expression of the HA‐tagged endogenous CLRN1 in retina homogenates from adult N‐HA *Clrn1* knock‐in mice. Retinas from C57BL/6J mice (P40) were collected as controls. The last two lanes show a duplicate experiment representing retina homogenates from two different P28 N‐HA *Clrn1* knock‐in mice. (B) Detection of CLRN1 in the HA‐tagged *Clrn1* mouse retina homogenates in the absence and presence of PNGase F enzyme. Note the shift in size towards a lower molecular mass in the presence of PNGase F. The C57BL/6J and *Clrn1* KO mouse retina extracts were used as negative controls. (C) Detection of endogenous CLRN1 in adult human retina (human retina lysates in two different buffers are shown). Note the characteristic shift in size following PNGase F enzymatic treatment. (D) Detection of AAV‐expressed N‐terminal HA‐tagged CLRN1 (main isoform) following subretinal delivery in C57BL/6J mice. Note: treatment with PNGase F resulted in a complete shift to a single lower‐molecular‐mass product, at the same size as endogenous HA‐tagged CLRN1 shown in B. Due to the low level of endogenous HA‐CLRN1, blots A, B, and C have a higher background and display nonspecific bands, which are also present in control C57BL/6J mice. In contrast, nonspecific bands are absent in blot D, due to the higher levels of recombinant AAV‐expressed CLRN1.

We first evaluated the endogenous CLRN1 expression by immunoblot analysis using homogenates of N‐HA *Clrn1* knock‐in mouse retinas and a commercially available rabbit monoclonal anti‐HA antibody (Figure 5). Previous *in vitro* biochemical assays have shown that CLRN1 contains a single N‐linked glycosylation site and undergoes a change in its molecular mass in the presence of PNGase F, an enzyme that removes all N‐linked oligosaccharide side chains 3, 9. In the experiments below, we took advantage of this characteristic shift in CLRN1 molecular mass following enzymatic deglycosylation and demonstrated CLRN1‐specific expression in retinal extracts by also including negative and positive controls. In the absence of PNGase F, we detected the presence of a specific HA‐tagged CLRN1 protein band of approximately 26 kDa in neural retina extracts from HA‐tagged *Clrn1* knock‐in mice (Figure 5A). Enzymatic treatment with PNGase F resulted in a complete shift of this band to a lower molecular mass, below 25 kDa, consistent with the predicted molecular size of the non‐glycosylated main CLRN1 isoform (Figure 5B). As expected, this band was absent from C57BL/6J wild‐type controls, as well as albino wild‐type A/J and *Clrn1* KO A/J mouse retinas. We analyzed retina extracts from N‐HA *Clrn1* knock‐in mice at various ages in order to investigate if CLRN1 protein was expressed continuously in the retina. We determined that CLRN1 expression persisted throughout adulthood, even in older animals with very low levels of *Clrn1* transcripts (supplementary material, Figure S7). We also generated a positive control sample by expressing the main *Clrn1* isoform in mouse retinas using a recombinant AAV vector. Retinal extracts were analyzed by immunoblotting at 4 weeks following subretinal vector delivery in C57BL/6J mice. The AAV‐expressed N‐terminal HA‐tagged CLRN1 produced several bands between 26 and 30 kDa, reflecting the various degrees in protein glycosylation (Figure 5D). Following enzymatic treatment with PNGase F, these bands collapsed into a single lower‐molecular‐mass product of approximately 22 kDa, representing the non‐glycosylated, recombinantly expressed CLRN1 main isoform, running at a similar size to the endogenous HA‐tagged CLRN1 from N‐HA‐*Clrn1* knock‐in retinas.

We next attempted to detect endogenous N‐HA CLRN1 protein in retina sections from our HA‐tagged *Clrn1* knock‐in mice by immunostaining. In spite of testing multiple anti‐HA antibodies and various tissue processing techniques, we were not able to obtain a specific signal in the HA‐tagged *Clrn1* knock‐in mice, similar to other researchers who tried to detect CLRN1 protein in the retina with anti‐CLRN1 antibodies 11. The staining distribution in the HA‐tagged *Clrn1* knock‐in mice was similar to the background signal in C57BL/6J controls, suggesting that the signal is below the detection limit of this assay, due to low levels of endogenous HA‐tagged CLRN1 and epitope masking effects (supplementary material, Figure S8). In contrast, recombinant N‐terminal HA‐tagged CLRN1 could successfully be detected with anti‐HA antibodies by immunostaining, either in transiently transfected HEK cells or in retinal tissue following intraocular AAV‐vector delivery in wild‐type C57BL/6J mice (supplementary material, Figure S9).

In the last set of experiments, we used immunoblotting to evaluate CLRN1 protein expression in human retina. Detection of CLRN1 in human retinal extracts is difficult, due to the presence of multiple nonspecific bands and the lack of negative controls. Indeed, we identified multiple bands in the 25–50 kDa range following immunoblotting using an anti‐CLRN1 rabbit polyclonal antibody (Figure 5C). In order to differentiate specific signals from other bands, we relied on the CLRN1 migration pattern identified in the above experiments with N‐HA‐*Clrn1* knock‐in retinas (Figure 5B). We identified a specific band corresponding to the endogenous human CLRN1 based on its characteristic shift towards a lower size following enzymatic treatment with PNGase F (Figure 5C). This migration pattern was consistent with the behavior of the main CLRN1 isoform. Taken together, these data provide evidence that in both mouse and human retinas, the main CLRN1 isoform exhibits continuous expression at the protein level, a result with important implications for developing AAV‐based gene therapy approaches to treat vision loss in USH3 patients.

## Discussion

Previous studies on CLRN1 localization have generated inconsistent results, reporting its presence in either the inner retina or/and photoreceptors. Our study resolves a long‐standing controversy and uncertainty in the field, and provides solid evidence that *Clrn1* mRNA in mouse, NHP, and human retinas is expressed in Müller glia and not photoreceptors. Our results extend the previous discovery that *Clrn1* mRNA is localized in Müller glia in the developing mouse retina by showing that a similar pattern of expression persists in adult retinas in distinct species 11. We also show that *CLRN1* is continuously expressed at the protein level in mouse and human retinas. These findings place Müller cells to the center of future mechanistic and therapeutic studies to prevent vision loss in USH3 disease.

The discrepancy in the ocular phenotype between USH3 patients and mouse models of USH3 disease remains a mystery. It is possible that there are different CLRN1 interaction partners among species, as well as distinct anatomical features of the Müller glia network in human retina. Müller glia play essential roles in photoreceptor development, function, and survival 26, 27, 28, 29, 30. In addition to providing structural support, Müller glia regulate the levels of glutamate neurotransmitter in the retina; process retinoids; and release neurotrophic factors 31, 32. At the OLM, they extend apical microvilli reaching between the photoreceptor inner segments and subretinal space. Specialized junctions at the OLM region interconnect neighboring photoreceptor neurons and Müller glia apical processes. Further studies and novel experimental systems are needed to establish which of these multiple functions of Müller cells are disrupted in USH3 patients and lead to pathology. As a tetraspanin, CLRN1 may function as a key regulator of cell adhesion and stabilize the Müller glia actin cytoskeleton and apical microvilli at the OLM region. CLRN1 may enable molecular links between Müller glia and photoreceptors by recruiting proteins involved in cell–cell adhesion and signaling at the plasma membrane of Müller cells. Identifying the precise subcellular localization of CLRN1 protein and that of its binding partners in the human and mouse retina may provide clues to understanding the phenotypic differences between the two species, and the mechanism by which mutations in *CLRN1* drive the disease progression in USH3 patients.

Interestingly, mutations in Crumbs homolog‐1 (CRB1), a protein essential for the integrity of adherens junctions and Müller glia–photoreceptor interactions, are associated with a wide spectrum of retinal dystrophies, including Leber congenital amaurosis (LCA) and early‐onset recessive retinitis pigmentosa (RP) 27, 33. CRB1 is found at the subapical region above the adherens junctions at the OLM, both in the inner segments of photoreceptors and in the apical microvilli of Müller glial cells 34. Mouse models of *Crb1* loss of function display defects in the OLM, focal laminar disorganization, and displacement of photoreceptor cells, a phenotype strongly dependent on the genetic background 35, 36, 37, 38, 39. In contrast, the laminar architecture of the retina in USH3 models is normal 11, 12. Moreover, the USH3 syndrome retinal phenotype in patients is different from that caused by *CRB1* mutations 2, 40, 41. This suggests that CLRN1 in Müller glia may impact photoreceptors through distinct mechanisms, independent from the Crumbs molecular complex. Detection of early pathological changes associated with the human USH3 retinal phenotype is key for understanding the specific roles of Müller glia‐expressed CLRN1 in photoreceptor development, structural stability, and function.

In summary, by combining the scRNA‐seq data analysis with a highly sensitive ISH assay, we were able to directly examine the pattern of *CLRN1* mRNA expression in different species, and provide a firm answer to the essential question regarding the cellular source of *CLRN1* in the adult human retina. These results are relevant for future studies addressing the biological significance of CLRN1 protein expression in Müller glia and its impact on photoreceptor cells, with the ultimate goal of developing safe therapeutic strategies to prevent vision loss in the USH syndrome.

## Author contributions statement

AD and LX wrote the manuscript, and conceived and designed the study. LX, SNB, CPS, FD, OA and YW carried out experiments, generated figures, and analyzed data. ESL, KAM, WWH, WCS, JTH, SB, JDA and AD interpreted data and revised the manuscript. All the authors agreed with the submission in its final form.

## Supporting information


**Supplementary materials and methods**

**Figure S1.** Detection of *Clrn1* mRNA in the mouse retinas during postnatal development and throughout adulthood
**Figure S2.** *Clrn1* mRNA localization in albino A/J mice (P40)
**Figure S3.** Dual RNAscope *Clrn1* ISH and glutamine synthetase (GS) immunohistochemistry in mouse retina (P8)
**Figure S4.** Detection of *CLRN1* transcripts in the human retina
**Figure S5.** Single‐cell RNAseq analysis of *Clrn1* expression in mice, NHP, and human retina
**Figure S6.** Characterization of the N‐terminal HA‐epitope‐tagged *Clrn1* knock‐in mouse model
**Figure S7.** Detection of the endogenous HA‐tagged CLRN1 protein in the HA‐tagged *Clrn1* knock‐in mice during postnatal development and adulthood
**Figure S8.** Immunofluorescence analysis of N‐HA‐*Clrn1* knock‐in retinas
**Figure S9.** Detection of recombinant HA‐tagged CLRN1 protein in HEK293 cells and mouse retina by immunohistochemistry
