# Supplementary material for: Clarin‐1 expression in adult mouse and human retina highlights a role of Müller glia in Usher syndrome
Source: J Pathol. 2019 Dec 4;250(2):195–204. doi: 10.1002/path.5360 (PMC7003947; doi:10.1002/path.5360)
Supplement: Supplementary file 1 — Supplementary materials and methods [file PATH-250-195-s001.docx]

Clarin-1 expression in adult mouse and human retina highlights a role of Müller glia in Usher syndrome

Xu, Bolch *et al. J Pathol* DOI: 10.1002/path.5360

**Supplementary materials and methods**

**Reference numbers refer to the main text list**

**Animal care and use**

All animal procedures, maintenance, and handling were approved by the University of Florida Institutional Animal Care and Use Committee (IACUC) and conducted in accordance with the Association for Research in Vision and Ophthalmology (ARVO) Statement for the Use of Animals in Ophthalmic and Vision Research (http://www.arvo.org/About/policies/statement-for-the-use-of-animals-in-ophthalmic-and-vision-research/). The *Clrn1* knock-in mouse with a hemagglutinin (HA) epitope-tagged CLRN1 was generated at the Jackson Laboratory (Bar Harbor, ME, USA) by injecting guide RNAs (sgRNA1: TGGCTTGGCATGACGAGAAA; sgRNA2: AAAAGATGATCTTCTTCTGC), Cas9, and donor template directly into C57BL/6J background mouse zygotes. The inserted 30 nucleotides, comprising the N-terminal HA tag sequence followed by an alanine, were confirmed by genotyping and DNA sequencing. PCR genotyping showed that DNA amplicons from C57BL/6J and the HA-tagged *Clrn1* knock-in homozygous mice migrate at the expected sizes of 419 and 449 bp, respectively. Sequencing the amplicon confirmed that DNA editing occurred in the correct position. Homozygous N-HA-tagged *Clrn1* knock-in mice were then distributed to Dr Astra Dinculescu at University of Florida and Dr Omar Akil at UCSF. The albino *Clrn1* knockout (KO) animals raised on an A/J background were a generous gift from Dr Imanishi (Case Western Reserve University). Wild-type albino A/J control mice were purchased from the Jackson Laboratory.

**RNAscope *in situ* hybridization**

Eyes were enucleated and fixed in 10% neutral buffered formalin for 20 h at 4 °C, then embedded in paraffin. Before staining, the paraffin was removed from 4-μm-thick sections using Dewax (Leica, Buffalo Grove, IL, USA), followed by immersion in 100% ethanol for 2 min. *Clrn1* transcripts were detected using an automated Leica Bond platform with heat-induced Leica ER2 antigen retrieval buffer solution and RNAscope 2.5 LS Protease III digestion (Advanced Cell Diagnostics, Hayward, CA, USA). After hybridization to probes described in the main text, a six-step amplification process was performed. Chromogenic detection was done using Fast Red (Advanced Cell Diagnostics). Slides were then counterstained with Gill’s hematoxylin for nuclear identification, dehydrated, and coverslipped using ClearVue XYL Mountant (Thermo Fisher Scientific, Waltham, MA, USA). The following tissue sections were used: wild-type C57BL/6J mouse retinas, *Clrn1* KO and wild-type mouse retinas on the A/J background, and retina sections from a 57-year-old human donor (a generous gift of Lions Eye Institute for Transplant & Research, Tampa, FL, USA). Images were collected with a Zeiss Axioplan 2 or a fully automated widefield DMi8 Leica fluorescence microscope.

**Single-cell RNA-seq library preparation and data analysis**

The mouse retina single-cell sequencing data were generated in-house at Duke University. The human retina single-cell sequencing data were obtained from an 86-year-old adult human donor, 3 h postmortem, and processed with papain digestion to dissociate cells. Retinas were collected from mice at postnatal day 60 (P60). Retinas were rapidly dissected and dissociated using papain digestion and mild trituration. Single-cell libraries were prepared following the manufacturer’s instructions (10X Genomics, Pleasanton, CA, USA). In brief, cell suspensions were loaded onto the 10X Chromium platform and barcoded using Chromium Single Cell 3′ v2 reagents. Libraries were sequenced with paired-end reads, with Read 1 (26 base pairs) and Read 2 (98 base pairs), on the NextSeq500 at the Genomics Resources Core Facility (High Throughput Center) at Johns Hopkins University (human data) or the Duke sequencing facility (mouse data). Raw sequence data were aligned using Cell Ranger software (10X Genomics) with the ENSMBL database mm10. The output of the software included read counts for levels of expression and t-distributed stochastic neighbor embedding (t-SNE) plots. The following genes were used to identify cell types: ganglion cells (*Pou4f2*, *Thy1*, *Nefl*), amacrine cells (*Tfap2a*, *Pax6*, *Gad1*), bipolar cells (*Vsx2*, *Grm6*, *Grik1*), horizontal cells (*Calb1*, *Lhx1*), rod photoreceptors (*Rho*, *Nrl*, *Nr2e3*), cone photoreceptors (*Opn1mw*, *Arr3*), astrocytes (*Pax2*, *S100b*), Müller glia (*Vim*, *Slc3a2*, *Glul*, *Rlbp1*), RPE (*RPE65*, *Lrat*), pericytes (*Tagln*, *Acta2*), and endothelial cells (*Tie1*, *Cldn5*). Information on single-cell human data set is available from Dr Handa. Single-cell mouse data are deposited at BioProject (#PRJNA578478).

**Genotyping procedure**

The genotype of the HA-tagged *Clrn1* knock-in mice was confirmed by using the following primers: forward, 5′-GACCATGCAGGCAATTTGGAAG-3′, and reverse, 5′-TGACGCGTTGACAAGCAGAG-3′. Diluted mouse tail lysates containing approximately 20 ng of genomic DNA were used as a template for PCR analysis in a 20 μl reaction. PCR conditions were 94 °C for 60 s, followed by 34 cycles of 94 °C for 15 s, 60 °C for 15 s, 72 °C for 10 s, and finally 72 °C for 5 min. MyTaq Red DNA polymerase (cat No BIO-21108; Bioline, Memphis, TN, USA) was utilized. Amplified products were separated on 3% agarose gels. The reactions amplified a 419 bp product in C57BL/6J mice and a 449 bp product in the N-HA knock-in mice, as mentioned above.

**Reverse-transcription PCR (RT-PCR)**

For RT-PCR analyses, fresh retinas were collected from C57BL/6J and N-HA *Clrn1* knock-in mice at different postnatal days (P10, P21, and P28), flash-frozen, and stored at −80 °C until processed. Total RNA was isolated using an RNAeasy Mini kit (cat No 74104; Qiagen, Germantown, MD, USA), according to the manufacturer’s instructions. Total RNA (1 μg) was reverse-transcribed with an iScript cDNA synthesis kit (cat No. 1708890; Bio-Rad, Hercules, CA, USA). RT-PCR was performed with specific primers to amplify different isoforms of *Clrn1*, as previously described [10]. Three forward primers were designed: from exon 1 (F1: TTTACCGAAGCCTTTTCTCG), exon 2 (F2: AGGTACTCTCTGTATGAGGACAA), and exon 3 (F3: TCTTCTCCATGATTCTTGTCGTCT). A reverse primer designed from exon 4 (R: GTGGCCAAAGGAAGTCCATA) was common to all isoforms. *Actb* (forward primer: ACCAACTGGGACGACATGGAGAA; reverse primer: CATGGCTGGGGTGTTGAAGGT) was used as internal positive control. PCR amplification was performed under the following conditions: 95 °C for 1 min, followed by 34 cycles of 95 °C for 30 s, 55 °C for 15 s, and 72 °C for 10 s. PCR products were separated on 2% agarose gels. The DNA marker Hyper Ladder^TM^ 100 bp was obtained from Bioline (cat no BIO-33056; Bioline).

**Spectral domain optical coherence tomography (SD-OCT) and electroretinography (ERG)**

For *in vivo* retinal imaging, spectral domain-OCT images were obtained using the Leica Bioptigen Envisu Spectral-Domain OCT (Leica Microsystems, Wetzlar, Germany) as previously described [42]. Scotopic ERGs were recorded from overnight dark-adapted mice using an Espion ColorDome™ instrument (Diagnosys LLC, Lowell, MA, USA) as previously described [43].

**Recombinant AAV construct preparation and intraocular delivery in mice**

The N-terminal HA-tagged *Clrn1* isoform 2 cDNA, driven by the ubiquitous smCBA (small chicken β-actin) promoter, was packaged into an AAV2 capsid serotype, as previously described [44]. Viral titer was determined by real-time qPCR. Subretinal or intravitreal injections were given to 6-week-old C57BL6/J mice under anesthesia. Each eye received 1 μl of the AAV vector at a titer of 1 × 10^12^ viral genome copies/ml, and the retinas were collected for immunoblotting analysis at 4 weeks after vector delivery.

**Transient transfection of plasmid DNA in HEK293 cells**

The AAV plasmid containing the above N-terminal HA-tagged *Clrn1* cDNA used for AAV2 packaging was also used to transfect human embryonic kidney (HEK) 293 cells. Slides were seeded with 150 000 cells and transfected with the jetPRIME Polyplus transfection kit (cat no 114-07; VWR, Radnor, PA, USA), according to the manufacturer’s instructions. One microgram of plasmid was mixed with 4 µl of jetPRIME reagent in 200 µl of jetPRIME buffer and added to the cell culture media. After incubation for 24 h, the cells were fixed with 4% PFA and assessed using IHC.

**Immunoblotting analyses**

A human retina from the Lions Eye Bank (68-year-old donor) was divided into two approximately equal parts. Each half was sonicated in 0.5 ml of buffer with three bursts of 10 s, with 30 s on ice between bursts. One half was sonicated in 1% Triton X-100 in PBS containing protease inhibitors, and the other half was sonicated in an RIPA buffer containing 0.15 m NaCl, 0.05 m Tris-HCl, 1% Triton X-100, 1% sodium deoxycholate, and 0.1% SDS, pH 7.4. All neural retinas from N-HA-*Clrn1* knock-in mice and C57BL/6J control mice were dissected in PBS with Roche protease inhibitors (cat no 04693116001; Millipore-Sigma, Burlington, MA, USA). Eyecups (including neural retina and posterior eyecup) were also collected from C57BL/6J mice that received AAV vectors expressing N-HA-CLRN1 following subretinal injection. Retinas were homogenized and lysed by sonication in 150 µl of RIPA buffer (cat no R0278; Millipore-Sigma) or in 1% Triton X-100 in PBS with protease inhibitors and centrifuged for 5 min. The supernatant was removed into a clean tube and protein concentration was measured with a NanoDrop ND-2000 spectrophotometer (Thermo Fisher Scientific, Wilmington, DE, USA). Supernatants were either left untreated or treated with the PNGase F enzyme kit (cat no P0704S; New England Biolabs, Ipswich, MA, USA) to remove the N-linked glycan side chain from CLRN1 protein. An 18 µl sample of mouse or human retina extract was mixed with 2 µl of Glycoprotein denaturing buffer, 4 µl of Glycobuffer 2, 4 µl of 10% Nonidet P40, 2 µl (1000 U) of PNGase F, and 10 µl of water, and incubated at room temperature for 1 h. Extracts of retina (up to 150 µg total protein) were loaded onto 4–20% Mini Protean TGX gradient gels (cat no 456-1094; Bio-Rad Laboratories, Hercules, CA, USA) and electrophoresed at 150 V for 50–75 min. Proteins were transferred to Immobilon-FL polyvinylidene fluoride (PVDF) membranes (cat no IPFL 00010; Millipore-Sigma) at 200 mA for 1 h and blocked in Odyssey blocking buffer (cat no 927-40000; LI-COR Biosciences, Lincoln, NE, USA) for 30 min. A rabbit monoclonal anti-HA antibody (cat no 3724S, clone C29F4, 1:3000; Cell Signaling Technology, Danvers, MA, USA) was added in a 1:1 solution of blocking buffer and PBS, and rocked gently overnight at room temperature. The membrane was washed twice in PBS with 0.05% Tween-20 for 5 min, rinsed briefly in PBS, and inclubated with the Invitrogen Goat anti-rabbit Alexa Fluor Plus 800 secondary antibody (cat no A32735, 1:15 000; Thermo Fisher Scientific, Waltham, MA, USA) for 1 h. The blot was washed twice in PBS with 0.05% Tween-20, rinsed in PBS, and then scanned on an Odyssey CLx infrared fluorescence imaging system (LI-COR Biosciences). The mouse monoclonal anti-alpha tubulin (cat no T5168, clone B-5-1-2, 1:5000; Millipore Sigma, Burlington, MA, USA) was used as a loading control antibody, followed by a secondary antibody IRDye 680RD Goat anti-mouse (cat no 925-68070, 1:15 000; LI-COR Biosciences). Immunoblotting was repeated at least four times using independent biological replicates.

**Immunofluorescence and immunohistochemistry**

Staining was performed on sections from either frozen or paraffin‐embedded eye tissue. For frozen sections, cornea and lens were gently removed in 4% PFA and fixed for 25 min, followed by immunofluorescence assays as previously described [45]. In brief, the eyecups were immersed in graded sucrose solutions of 10%, 20%, and 30% in PBS at 4 °C for at least 30 min in each. Dissected eyecups were then incubated in optimal cutting temperature (OCT) medium (Fisher, cat no 4585) for 30 min at room temperature, and placed in base molds filled with OCT, then frozen rapidly in liquid nitrogen. Cryosections were collected on SuperFrost slides (cat no 48311-703; VWR) using a Leica CM 3050S cryostat. In brief, 12- to14-µm frozen sections were cut onto histology slides and pretreated with Basic (pH 10.0) Antigen Retrieval Reagent (cat no CTS016; R&D Systems, Minneapolis, MN, USA) at 80 °C for 5 min, followed by blocking with PBS with 0.1% Triton X-100 and 10% donkey serum for 1 h. Slides were then incubated with the primary antibody in PBS with 0.1% Triton X-100 and 2% donkey serum for 1 h, washed three times in PBS, then incubated with secondary antibodies for 30 min. Sections were mounted with Fluoromount-G (SouthernBiotech, Birmingham, AL, USA). The following primary antibodies were used: high affinity Roche anti-HA rat monoclonal (cat no 11867423001, clone 3F10, 1:50; Millipore-Sigma), mouse monoclonal anti-CTBP2 (cat no 612044, clone 16BD, 1:1000; BD Biosciences, San José, CA, USA), and rabbit anti-Cav1.4 (1:1000; a generous gift from Dr Amy Lee [46]). Secondary antibodies were Invitrogen anti-rat Alexa Fluor 488 (cat no 11006, 1:500; Thermo Fisher Scientific, Waltham, MA, USA), anti-mouse Alexa Fluor 594 (cat no A21155, 1:500; Thermo Fisher Scientific), and anti-rabbit Alexa Fluor Plus 680 (cat no A32802, 1:500; Thermo Fisher Scientific).

Eyes were also processed into paraffin blocks, and 4-μm paraffin sections were collected for immunofluorescence and immunohistochemistry. Sections were blocked with 1% BSA in PBS for 1 h at room temperature, then incubated with primary antibody overnight at room temperature and washed three times in PBS, followed by secondary antibody for 1 h. Primary and secondary antibodies were diluted either in blocking buffer with 0.5% Triton X-100 or in PBS alone. The following primary antibodies were used in this experiment: rabbit monoclonal anti-HA (cat no 3724S, clone C29F4, 1:300; Cell Signaling Technology), Roche monoclonal rat anti-HA (cat no 11867423001, clone 3F10, 1:200; Thermo Fisher Scientific), and anti-HA fluorescein-conjugated (cat no 11988506001, 1:200; Thermo Fisher Scientific). The secondary antibodies were Invitrogen Alexa Fluor 594 anti-rat (cat no 11007; Thermo Fisher Scientific), or Alexa Fluor 594 anti-rabbit (cat no A11012; Thermo Fisher Scientific), at 1:500 dilution. Slides were coverslipped and mounted with Prolong Diamond Antifade Mountant with DAPI (cat no P36962; Thermo Fisher Scientific). For overlaying the signals of *Clrn1* transcript as detected by the RNAscope *in situ* hybridization with the Müller cell protein marker glutamine synthetase, immunohistochemistry experiments with HRP staining were run on a Leica Bond Rx Automated Stainer. First, heat-induced epitope retrieval steps were performed at 100 °C for 20 min, followed by ISH, as described above. Subsequently, the same ocular tissue sections were used for IHC. The slides were dried, incubated in peroxidase blocking solution for 10 min at room temperature, and stained for the Müller cell marker by incubation with an anti-glutamine synthetase rabbit polyclonal primary antibody (cat no ab73593, 1:300; Abcam, Cambridge, MA, USA) for 30 min at room temperature. The IHC signal was detected using the Bond polymer refine detection system (cat no DS9800; Leica Biosystems, Buffalo Grove, IL, USA). Images were acquired with a fully automated widefield DMi8 Leica fluorescence microscope.

**Mouse auditory brainstem response (ABR)**

The ABR assay was performed using a Tucker Davis Technologies System III workstation (Alachua, FL, USA). Mice were anesthetized by intraperitoneal injection of a ketamine (100 mg/kg)/xylazine (10 mg/kg) cocktail. Anesthetized mice were then placed on a heating pad, and electrodes were placed subcutaneously in the vertex, underneath the left or right ear, and on the back near the tail. Tone stimuli of 4, 5.6, 8, 11.2, 16, 22, 32, and 45.3 kHz were calibrated with a precision microphone system (PS9200 Kit; ACO Pacific, Belmont, CA, USA), using the TDT SigCal software package (Tucker Davis Technologies). The recorded signals were band-pass filtered (300 Hz to 3 kHz) and amplified 100 000 times. The number of acquisition trials was set to 500 averages. Maximum stimulus intensity was set to 95 dB peak SPL, with attenuation decreasing from 85 dB to 0 dB SPL at 5 dB intervals. Band-pass filters (500–3000 Hz) were applied to the traces before analysis.
