# Supplementary material for: Clarin‐1 expression in adult mouse and human retina highlights a role of Müller glia in Usher syndrome
Source: J Pathol. 2019 Dec 4;250(2):195–204. doi: 10.1002/path.5360 (PMC7003947; doi:10.1002/path.5360)
Supplement: Supplementary file 2 — Figure S1. Detection of Clrn1 mRNA in the mouse retinas during postnatal development and throughout adulthood Figure S2. Clrn1 mRNA localization in albino A/J mice (P40) Figure S3. Dual RNAscope Clrn1 ISH and glutamine synthetase (GS) immunohistochemistry in mouse retina (P8) Figure S4. Detection of CLRN1 transcripts in the human retina Figure S5. Single‐cell RNAseq analysis of Clrn1 expression in mice, NHP, and human retina Figure S6. Characterization of the N‐terminal HA‐epitope‐tagged Clrn1 knock‐in mouse model Figure S7. Detection of the endogenous HA‐tagged CLRN1 protein in the HA‐tagged Clrn1 knock‐in mice during postnatal development and adulthood Figure S8. Immunofluorescence analysis of N‐HA‐Clrn1 knock‐in retinas Figure S9. Detection of recombinant HA‐tagged CLRN1 protein in HEK293 cells and mouse retina by immunohistochemistry [file PATH-250-195-s002.doc]

**Clarin-1 expression in adult mouse and human retina highlights a role of Müller glia in Usher syndrome**

Xu, Bolch *et al. J Pathol* DOI: 10.1002/path.5360

**
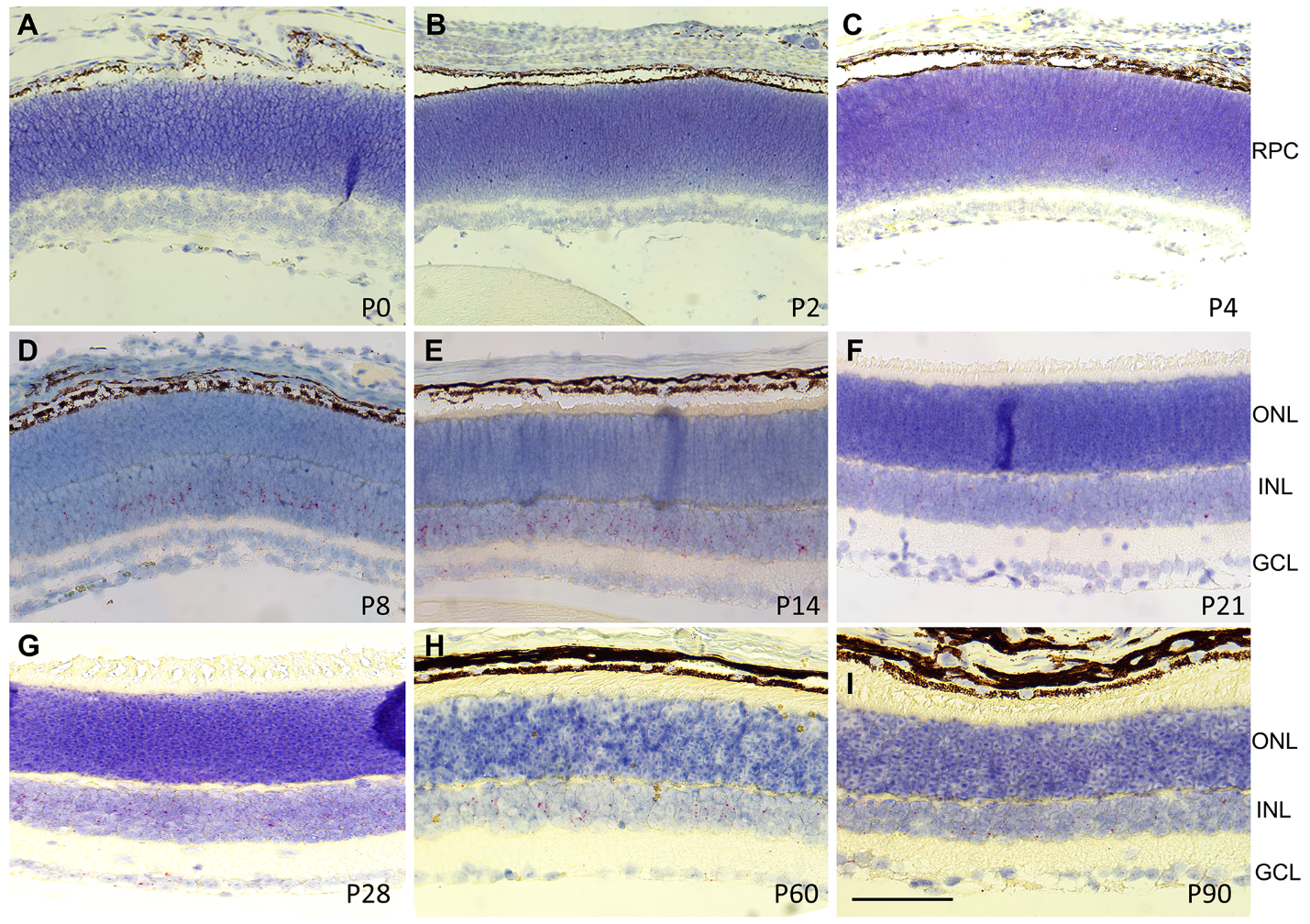
**

**Figure S1. Detection of *Clrn1* mRNA in the mouse retinas during postnatal development and throughout adulthood.** Representative images showing *Clrn1* transcripts at (A) P0, (B) P2, (C) P4, (D) P8, (E) P14, (F) P21, (G) P28, (H) P60, and (I) P90. The ISH signal is weakly visible at P4 and increases in intensity at P8 up until P14. RPC, retinal progenitor cells; ONL, outer nuclear layer; INL, inner nuclear layer; GCL, ganglion cell layer. Scale bar: 50 µm.

**
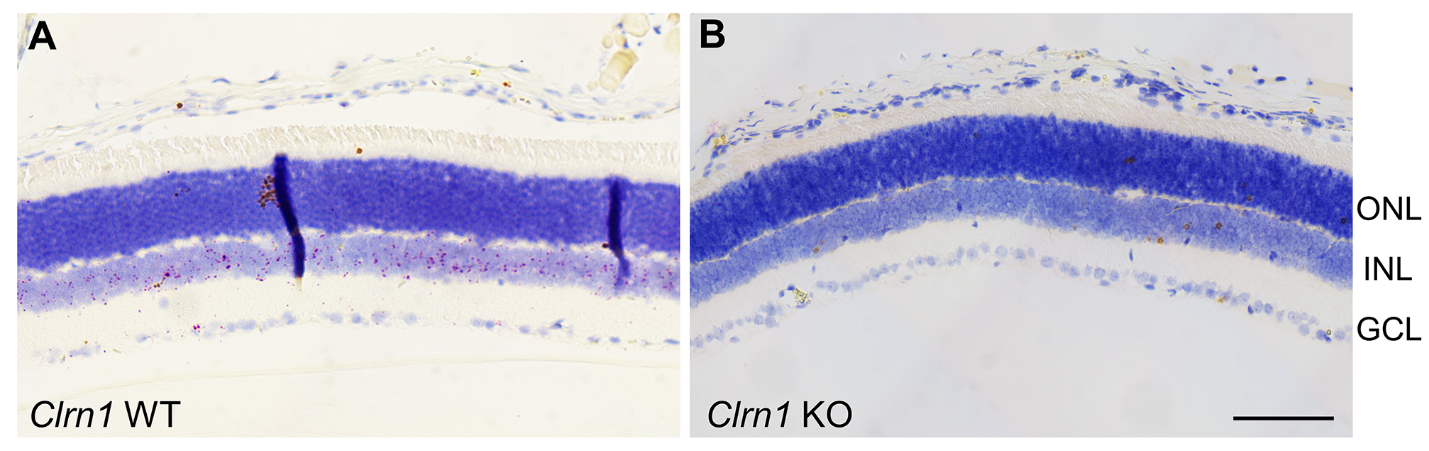
Figure S2. *Clrn1* mRNA localization in albino A/J mice (P40).** (A) Wild-type retina. (B) *Clrn1* KO retina. Note the red punctate signals representing *Clrn1* mRNA in the inner nuclear layer in WT mice only. ONL, outer nuclear layer; INL, inner nuclear layer; GCL, ganglion cell layer. Scale bar: 50 µm.


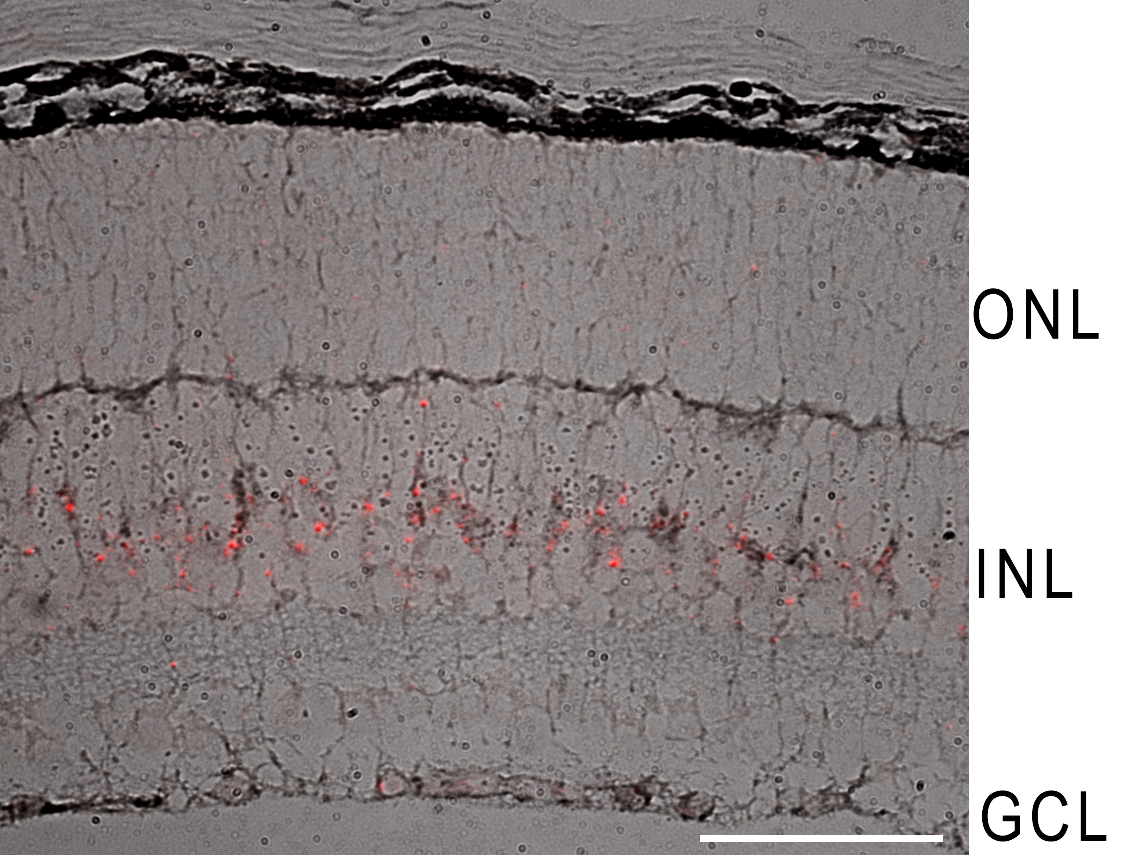


**Figure S3. Dual RNAscope *Clrn1* ISH** **and glutamine synthetase (GS) immunohistochemistry in mouse retina (P8).** *Clrn1* transcripts (red fluorescent dots) co-localize with GS staining in the middle of the INL near the Müller cell bodies (dark gray color). ONL, outer nuclear layer; INL, inner nuclear layer; GCL, ganglion cell layer. Scale bar: 50 µm.

**
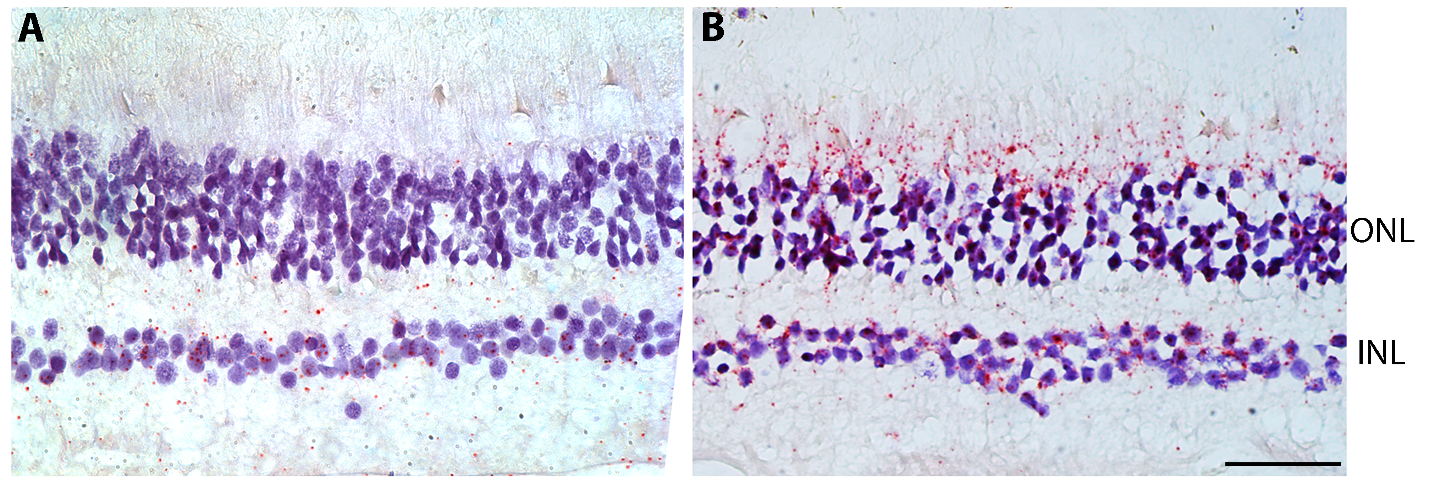
Figure S4. Detection of *CLRN1* transcripts in the human retina.** (A) *CLRN1* mRNA (red dots) is predominantly found in the inner nuclear layer. (B) Positive control using a *POLR2A* probe. Note expression in the inner segment, outer nuclear layer, and inner nuclear layer. ONL, outer nuclear layer; INL, inner nuclear layer. Scale bar: 50 µm.


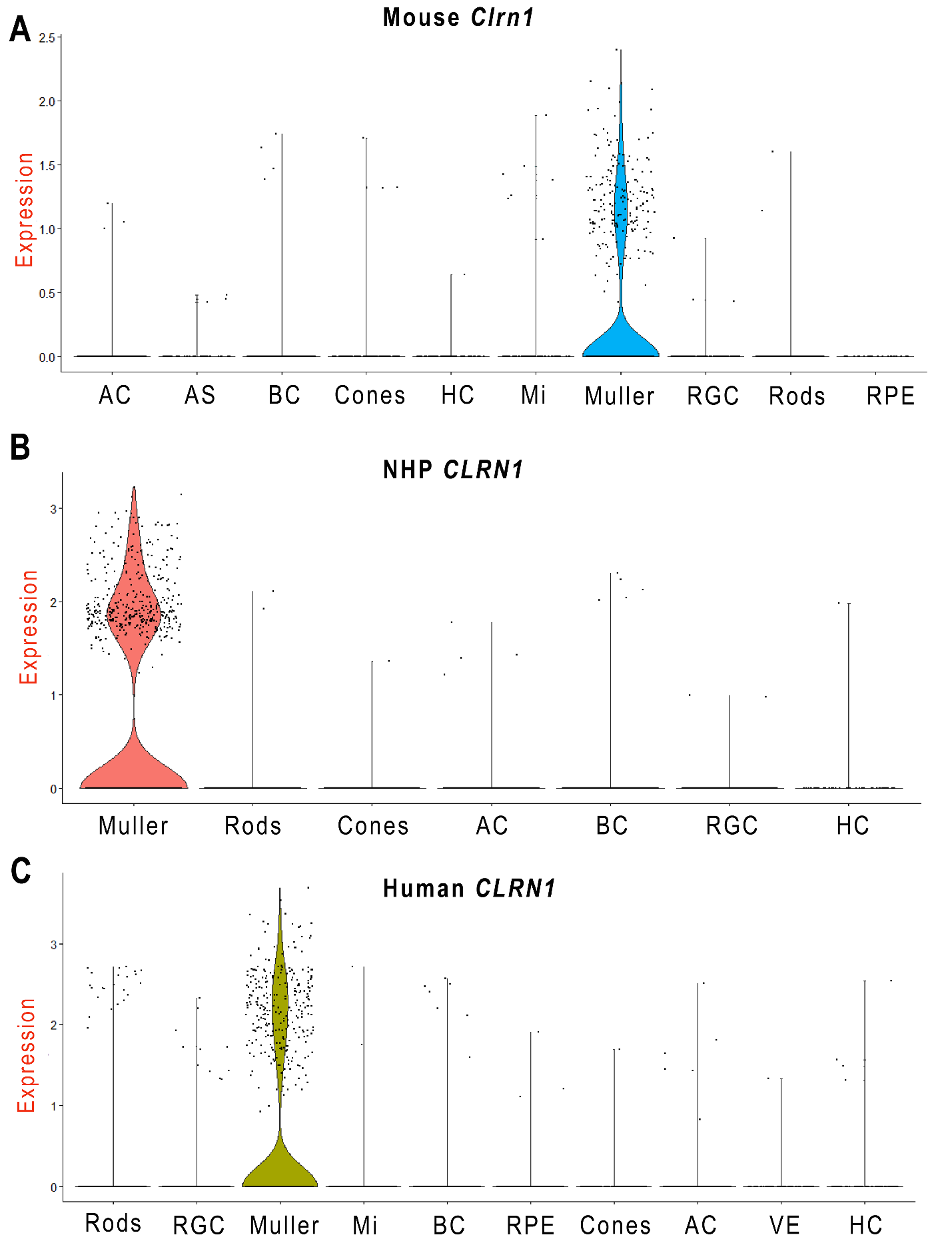


**Figure S5. Single-cell RNAseq analysis of *Clrn1* expression in mouse, NHP, and human retina.** Using Seurat, we extracted the expression of *Clrn1* in all cell clusters. The violin plots show cell types with *Clrn1-*enriched expression in (A) mouse, (B) non-human primate (NHP), and (C) human retinas. Cell populations are: Müller glia (Muller), rods, cones, retinal ganglion cells (RGC), horizontal cells (HC), bipolar cells (BC), amacrine cells (AC), microglia (Mi), vascular endothelial (VE), retinal pigment epithelium (RPE), and Astrocytes (AS).

**
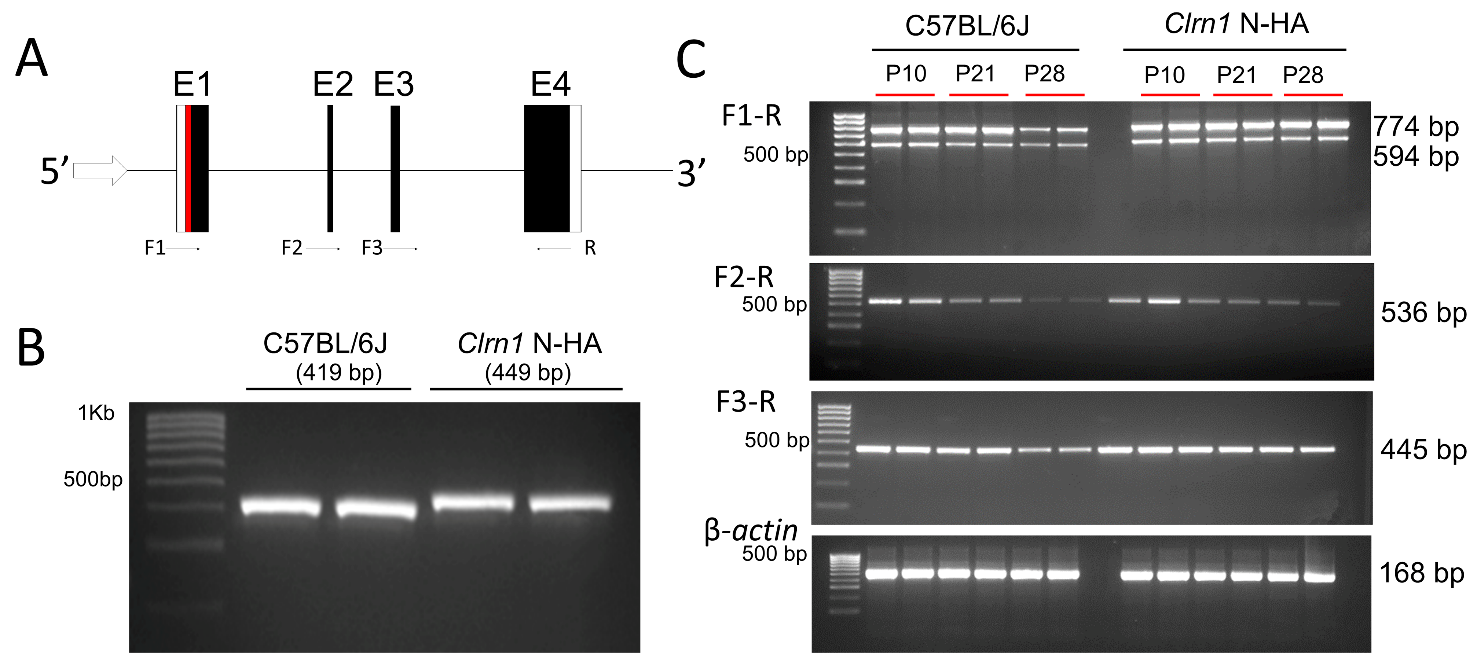
**

**Figure S6. Characterization of the N-terminal HA-epitope-tagged *Clrn1* knock-in mouse model.** (A) Schematic illustration of the mouse *Clrn1* gene with its four exons (rectangles) and introns (lines). The red box indicates the HA-tag insertion, right after the ATG start codon. (B) PCR genotyping of tail DNA from the C57BL/6J mice and N-HA tagged *Clrn1* knock-in mice. (C) Detection of *Clrn1* mRNA in the mouse retina by RT-PCR. Bands were cut, purified, and sequenced to confirm the expression of different *Clrn1* isoforms in the retina. Three forward primers, F1, F2, F3, from exons 1, 2, and 3, respectively, and one reverse primer R from exon 4, were used in the reaction, as indicated in A. PCR products were loaded to four different 2% agarose gels in the order as indicated: C57BL/6J and *Clrn1* N-HA at P10, P21, and P28, respectively. Using the F2 primer against exon 2, we also found an amplicon containing exons 2, 3, and 4. Using the F3 primer against exon 3, we found an amplicon containing exons 3 and 4. However, by using primers F1/R, we did not detect the full-length *Clrn1* isoform 1 containing exons 1, 2, 3, and 4 (encoding a 250-amino acid protein), suggesting that isoform 2 is preferentially amplified with this primer pair. All transcripts were confirmed by sequence analysis. *β*-*Actin* (*Actb*)was used as an internal loading control.


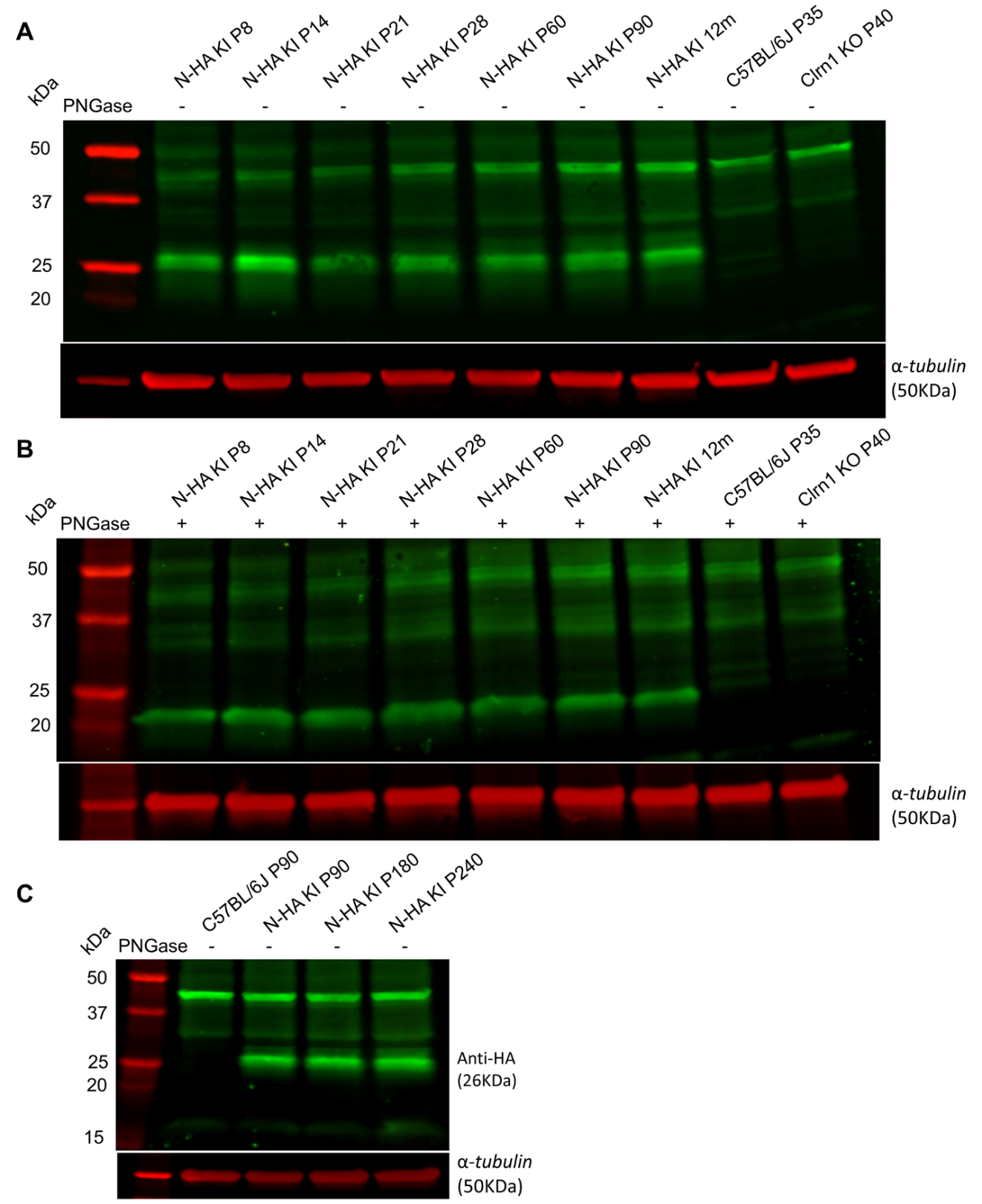


**Figure S7.** **Detection of the endogenous HA-tagged CLRN1 protein in the HA-tagged *Clrn1* knock-in mice during postnatal development and adulthood.** (A) Representative immunoblots showing the expression of the HA-tagged endogenous CLRN1 in retina homogenates from various ages, from postnatal to adult stages, adult N-HA-*Clrn1* knock-in mice. Retinas from C57BL/6J mice and *Clrn1* KO were collected as controls. (B) Same samples as in A, treated with PNGase F enzyme. The C57BL/6J and *Clrn1* KO mouse retinal extracts were used as negative controls. (C) CLRN1 protein detection at P90, P180, and P240, showing that it is continuously produced in adult stages.

**
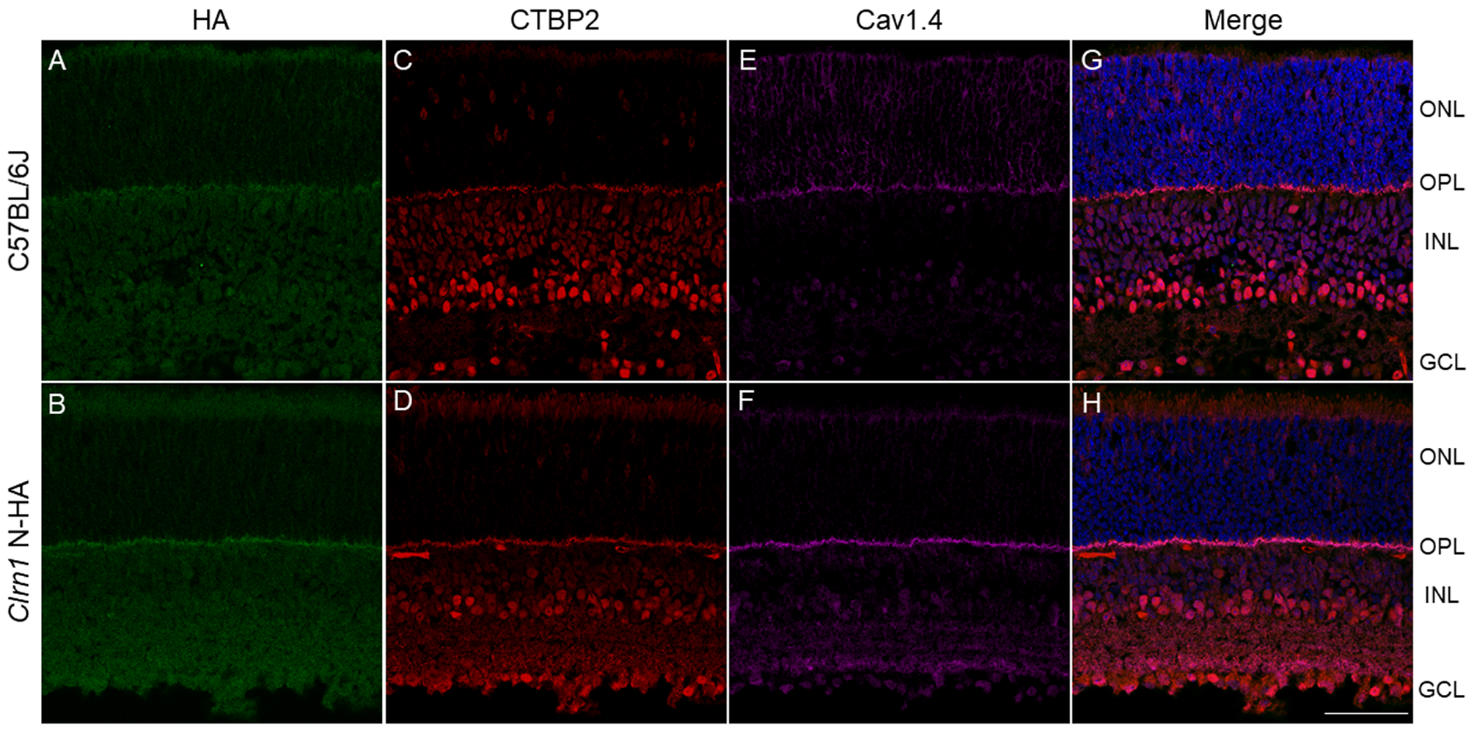
Figure S8. Immunofluorescence analysis of N-HA-*Clrn1* knock-in retinas.** HA-CLRN1 (green), CTBP2 (Ribeye, red), voltage-gated calcium channel (Cav1.4, violet), and DAPI (blue). ONL, outer nuclear layer; OPL, outer plexiform layer; INL, inner nuclear layer; IPL, inner plexiform layer; GCL, ganglion cell layer. Scale bar: 50 µm.


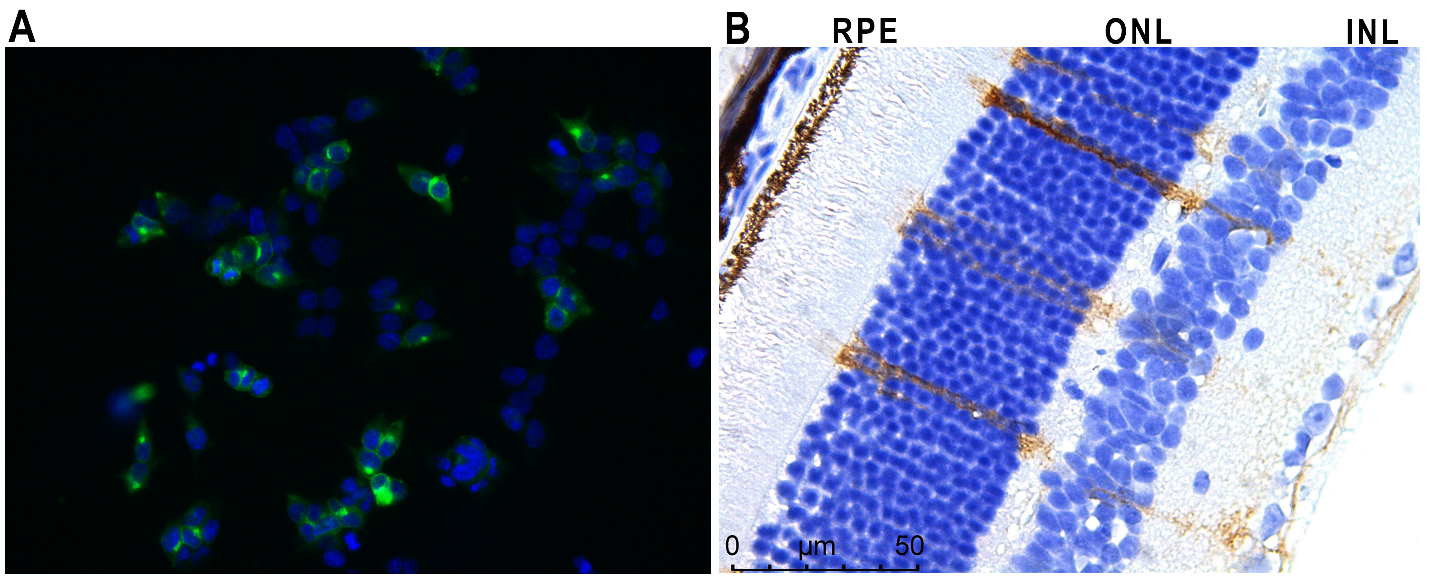


**Figure S9. Detection of recombinant HA-tagged CLRN1 protein in HEK293 cells and mouse retina by immunohistochemistry.** (A) HEK293 cells were transiently transfected with N-terminal HA-tagged CLRN1, and the HA signal was detected with anti-HA-fluorescein-conjugated antibody (green). (B) Detection of AAV-expressed CLRN1 protein in the mouse retina. Note the presence of AAV-expressed CLRN1 in radial Müller glia cells across the retina (brown). RPE, retinal pigment epithelium; ONL, outer nuclear layer; INL, inner nuclear layer.
